# Plasma Levels of Heat Shock Protein 90 Alpha Associated With Colorectal Cancer Development

**DOI:** 10.3389/fmolb.2021.684836

**Published:** 2021-07-08

**Authors:** Wene Wei, Jiahui Zhou, Lipeng Chen, Haizhou Liu, Fuyong Zhang, Jilin Li, Shufang Ning, Shirong Li, Chen Wang, Yi Huang, Chang Zou, Litu Zhang

**Affiliations:** ^1^ Department of Research, Guangxi Medical University Cancer Hospital, Nanning, China; ^2^ Shenzhen People’s Hospital, The Second Clinical Medical College, Jinan University, The First Affiliated Hospital, Southern University of Science and Technology, Shenzhen, China; ^3^ Department of Laboratory, The First Affiliated Hospital of Guangxi Medical University, Nanning, China

**Keywords:** heat shock protein 90 alpha, colorectal cancer, metastasis, diagnosis, prognosis

## Abstract

**Aim:** The role of plasma heat shock protein 90 alpha (HSP90α) in colorectal cancer patients remains unclear. This study aimed to evaluate the relationship between HSP90α and the occurrence and development of colorectal cancer through diagnosis and prognosis value.

**Methods:** 635 colorectal cancer patients and 295 healthy controls were recruited. The HSP90α was measured by using the ELISA kit in all objects and the immune cells and common biomarkers as CEA, AFP, CA125, CA153 and CA199 were measured in all colorectal cancer patients. The relationship between plasma HSP90α with clinical features, common tumor markers and immune cells were also conducted. The survival analysis endpoint was progression-free survival (PFS).

**Results:** The levels of plasma HSP90α were significantly higher in colorectal cancer patients compared to healthy controls [51.4 (ng/ml) vs. 43.7 (ng/ml), *p* < 0.001]. In additional, the levels of plasma HSP90α were associated with gender and disease progress as stage, lymphatic and distant metastasis. Furthermore, plasma HSP90α was closed correlation with CEA, CA125, CA199 and percentage of B cells. However, the initial expression level of plasma HSP90α failed to show a prognostic value for progression-free survival in colorectal cancer.

**Conclusion:** The plasma Hsp90α was remarkable higher in colorectal cancer and correlated with common tumor biomarkers and immune cells. Plasma Hsp90α levels were associated with disease progress but a poor diagnosis performance and also failed to show a prognostic value in colorectal cancer.

## Introduction

Colorectal cancer (CRC) is one of the most common tumors worldwide. Approximately 1.8 million people were diagnosed with colorectal cancer and 861,000 died from colorectal cancer in 2018, which equates to 4,931 people diagnosed with colorectal cancer and 2,358 people dying from colorectal cancer every day (Bray et al., 2018). Metastasis and recurrence have always been difficult points in cancer treatment. The liver is the most common site of distant metastases and accounts for 20% of CRC patients who arrive in hospitals with synchronous disease, and approximately 50% of CRC patients develop liver metastases at some point in the course of their disease (Leonard et al., 2005; Manfredi et al., 2006). With the development of medical technology, the treatments for colorectal cancer patients include endoscopic and surgical local excision, downstaging preoperative radiotherapy and systemic therapy, extensive surgery for locoregional and metastatic disease, local ablative therapies for metastases, and palliative chemotherapy, targeted therapy, and immunotherapy (Hurwitz et al., 2004; André et al., 2009; Van Cutsem et al., 2009; Haller et al., 2011; Ahlenstiel et al., 2014; Baratti et al., 2016; Ma et al., 2017; Abdel-Rahman and Cheung, 2018). Distant metastatic CRC (mCRC) patients have historically been associated with poor survival. Without treatment, the median survival time of mCRC patients is < 12 months, and the 5 years survival rate is <10% (Mohammad and Balaa, 2009; Morris and Treasure, 2018). Undergoing bevacizumab in combination with oxaliplatin or capecitabine chemotherapy therapy, patients with colorectal cancer have a significantly improved prognosis, with a median progression-free survival (PFS) of approximately 9 months (Saltz et al., 2008; Cunningham et al., 2013). Patients with metastatic colorectal cancer have a good survival benefit from prompt and aggressive treatment. Therefore, timely detection of metastasis is particularly important for colorectal cancer patients.

Traditionally, computed tomography (CT) scan is the common modality for initial diagnosis of distant metastasis and the modality of choice in order to discover those patients for evidence of a recurrence of the disease (Benson et al., 2013). However, preoperative chemotherapy or neo-adjuvant therapy would generally decrease the sensitivity of CT scans as a method for monitoring metastasis in CRC patients (van Kessel et al., 2012; Rojas Llimpe et al., 2014). Therefore, an optimal strategy for early detection and diagnosis distant metastasis for CRC patients is essential. Meanwhile, an appropriate way to assess prognosis is also urgently needed.

Due to the accessibility and noninvasiveness advantages, peripheral blood samples are commonly used for biomarker determinations and are widely accepted as an acceptable intervention by patients and health workers. In addition to commonly used tumor markers such as CEA, AFP, CA125, CA153 and CA199, heat shock protein 90 alpha (HSP90α) is a broad-spectrum tumor marker worthy of attention in recent years. HSP90α is an intracellular molecular chaperone which is exposed to the extracellular space. It has been documented that the overexpression of HSP90α was associated with tumor development, progression, invasiveness, metastatic potentials and chemo-resistance in various types of cancers (Passarino et al., 2003; Eustace et al., 2004; Tsutsumi et al., 2008; Chang et al., 2009; Zuehlke et al., 2015). Previous studies showed that HSP90α levels with or without AFP can act as excellent diagnostic markers for liver cancer (Fu et al., 2017; Wei et al., 2020). In addition, HSP90α also showed a good diagnostic performance for lung cancer and early CRC patients (Shi et al., 2014; Kasanga et al., 2018). In China, HSP90α was approved for clinical application as a broad-spectrum tumor marker in 2016. However, the diagnostic and prognostic efficacies of plasma HSP90α in patients with colorectal cancer has not been thoroughly confirmed. In the current study, we will assess the diagnostic and prognostic value of HSP90α for CRC patients.

## Material and Methods

### Patients

635 CRC patients were recruited from the Department of Gastrointestinal Surgery, Guangxi Medical University Cancer Hospital from 1, Jan, 2018 to 31, Aug, 2019 with a median age 60 years. The inclusion criteria for all patients that there was: 1) confirmation of CRC by clinical manifestation and histopathological examination associated with imaging diagnosis; 2) the state of distant metastases was assessed; 3) no anti-tumor treatment or surgical resection were performed at the time of diagnosis; 4) the tumor markers, immune cells and HSP90α in peripheral blood were evaluated before any treatment; 5) complete information for clinical features were available. The exclusion criteria for all patients were if they had: 1) CRC combined with other cancers; 2) a history of malignant tumors in other organs; 3) radiation or chemotherapy prior to admission; 4) distant metastases which could not be evaluated. The control group included 170 patients from The First Affiliated Hospital of Guangxi Medical University who had received health examinations during the same period with a median age 37.5 years. Stages for patients with colorectal cancer were classified according to the American Joint Committee on Cancer Classification (the 7th edition). Patients were followed up for tumor assessments every 12 weeks. Disease progression assessed by using RECIST 1.1 (Eisenhauer et al., 2009).

### Methods

Detection of peripheral blood markers was performed as follows. Fasting elbow venous blood was collected in EDTA anticoagulant tubes to prepare plasma samples and in dried tubes to prepare serum samples. All samples were separated by centrifugation at 3,000 rpm for 10 min. The levels of plasma HSP90α were measured by using the ELISA kit for HSP90α protein (Yantai Protgen Biotechnology Development Co., Ltd., Yantai, China). All operations followed the manufacturer’s instructions. The kit was pre-incubated at 37°C for 30 min and the plasma samples were diluted 20 times with diluent solution provided in the kit. The standards were loaded together with the quality controls and the prepared samples (50 μL of each) added into 96-well plates followed by addition of 50 μL of anti–Hsp90aHRP-conjugated antibody. Then, the plate was incubated at 37°C for 1 h after gentle shaking. Next, the color reaction step was carried out after six washes. 50 μL of peroxide and 50 μL of 3, 3′, 5, 5′ -tetramethylbenzidine solution was added and the mixture was incubated at 37°C for a further 20 min followed by termination of the reaction with an acid stop buffer. Finally, the optical density was measured using a spectrophotometer at 450 nm for the detection wavelength, with 620 nm as the reference wavelength. The concentration of HSP90α protein in each sample was calculated according to a standard curve of optical density values. Meanwhile, the expression status of HSP90α in colon adenocarcinoma (COAD) was also evaluated by using data retrieved from The Cancer Genome Atlas (TCGA).

The levels of serum CEA, AFP, CA125, CA153 and CA19-9 were measured by chemiluminescence microparticle immuno assays (CMIA) using the Architect i2000SR analyzer and the corresponding reagent kits which were purchased from Architect Diagnostics, America. All operations followed the manufacturer’s instructions. The immune cells in this study including T cells, helper T lymphocytes (Th cells), suppressor T lymphocytes (Ts cells), Natural killer cells (NK cells) and B cells were defined as CD45+/CD3+ lymphocytes, CD45+/CD3+/CD4+ lymphocytes, CD45+/CD3+/CD8+ lymphocytes, CD45+/CD3-/CD16+/CD56 + lymphocytes and CD45+/cCD19 + lymphocytes, respectively. Flow cytometry (BD Biosciences, Franklin Lakes, NJ, United States) was performed to detect labeled cells and analyze the results.

### Statistical Analysis

Levels of the HSP90α, CEA, AFP, CA125, CA153 and CA19-9 were assessed by SPSS STATISTICS 23.0 (IBM, Chicago, IL, United States) to ascertain normal distribution and the data are presented as medians and ranges. Nonparametric Kruskal-Wallis H-test was used to compare the differences between groups. Wilcoxon rank sum test was used to analyze HSP90α expression in COAD samples from The Cancer Genome Atlas (TCGA). Correlation between the indicators was analyzed using Pearson analysis. The diagnostic value was analyzed using receiver operating characteristic (ROC) curves, with the area under the curve (AUC). The optimal cut-off values for ROC curves were established using the Youden Index (YI = sensitivity + specificity −1). Progression-free survival (PFS) was the primary study end point, and was defined as the time from initial diagnosis to the date of disease recurrence and was censored at the last follow-up or at the time of death from any cause. The survival curves were generated using the Kaplan-Meier curve and survival differences were estimated by a log-rank test. *p* < 0.05 was considered statistically significant.

## Results

### Basic Profile of Tumor Markers and Immune Cells in Patients With Colorectal Cancer

**FIGURE 1:**
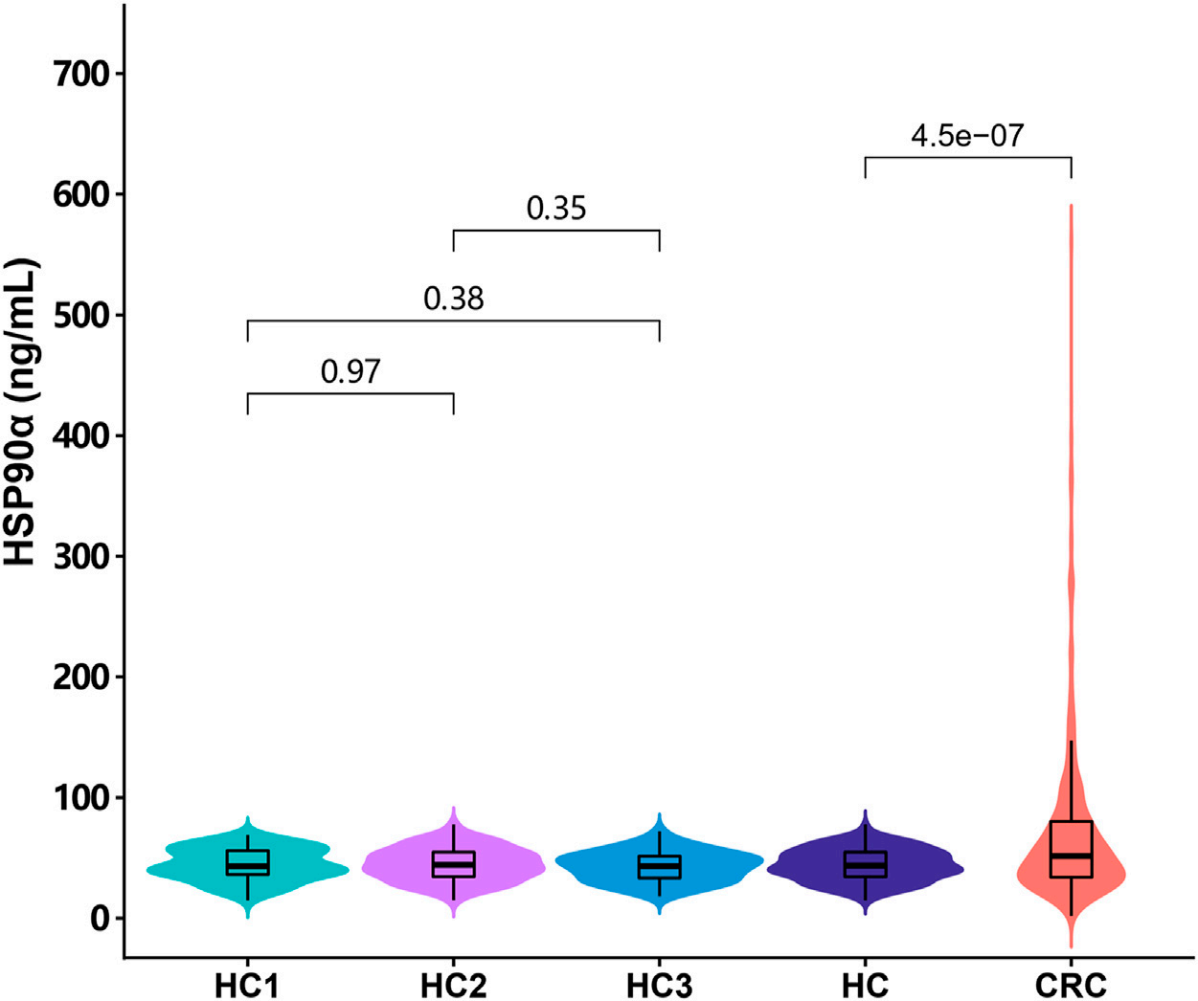
The distribution of the plasma HSP90α protein expression in colorectal cancer patients and healthy controls. HC1: subgroup under 40 years of age in healthy control group; HC2: subgroup of healthy controls aged 40–59 years; HC3: subgroup over 60 years of age in healthy control group; HC: all healthy controls, CRC: colorectal cancer.

This study recruited 635 cases of colorectal cancer patients and 295 cases of healthy controls. Routine tumor markers and immune cells were assessed in all patients and the results were showed in Table 1. The plasma levels of HSP90α protein were significantly higher in patients with colorectal cancer than healthy controls [51.4 (33.8, 80.3) ng/ml vs. 43.7 (34.3, 54.8) ng/ml; *p* < 0.001, Figure 1]. There was no significant difference in plasma HSP90α protein levels among healthy controls with different age distribution (all *p > 0.05*, Figure 1).

**TABLE 1:** Basic profile of tumor markers and immune cells in patients with colorectal cancer.

Parameters	Colorectal cancer	Healthy control	*p* value
CEA (ng/ml)	5.3 (2.4, 17.8)	0.9 (0.6, 1.4)	<*0.001*
AFP (ng/ml)	2.5 (1.9, 3.4)	2.1 (1.6, 2.8)	<*0.001*
CA125 (U/ml)	11.1 (7.7, 18.7)	11.7 (8.3, 17.2)	*0.952*
CA153 (U/ml)	9.3 (6.2, 13.3)	16.0 (13.0, 22.7)	<*0.001*
CA199 (U/ml)	9.1 (3.5, 41.4)	12.9 (7.4, 17.9)	*0.090*
HSP90α (ng/ml)	51.4 (33.8, 80.3)	43.7 (34.3, 54.8)	<*0.001*
TK1 (ng/ml)	0.8 (0.4, 1.3)	*—*	*—*
T cells (%)	66.1 (58.7, 72.13)	*—*	*—*
Th cells (%)	39.7 (33.9, 45.5)	*—*	*—*
Ts cells (%)	19.3 (15.2, 24.2)	*—*	*—*
Ratio	2.0 (1.6, 2.7)	*—*	*—*
NK (%)	13.1 (8.7, 18.8)	*—*	*—*
B cells (%)	12.0 (9.3, 15.7)	*—*	*—*

Ratio: the ratio of the Th cells to Ts cells. All values are presented in median and quartile intervals. The *p* values were shown in italics.

### Correlations Between the Plasma Levels of HSP90α Protein and Clinic-Pathological Characteristics in Colorectal Cancer Patients

The associations of plasma levels of HSP90α protein and clinic-pathological characteristics in colorectal cancer patients were showed in Table 2. Male patients with colorectal cancer were showed a significantly higher plasma level of HSP90α protein (*p = 0.002*). Meanwhile, the plasma levels of HSP90α protein were correlated with disease stage, lymphatic metastasis, and distant metastasis (all *p < 0.001*). There was no difference in plasma HSP90α protein expression among different pathological differentiation levels (*p = 0.153*).

**TABLE 2:** Correlations of the plasma HSP90α with clinicopathologic features in colorectal cancer.

Parameters	Frequency	HSP90α (ng/ml)	*p* value
Age (<60 years, ≥60 years)	315/320	51.4 (33.8, 80.3)/48.2 (32.5, 72.6)	*0.073*
Gender (male, female)	414/221	54.5 (35.9, 84.7)/43.7 (30.2, 71.7)	***0.002***
Differentiation (well, moderately, poor)	25/545/65	48.1 (31.0, 72.3)/50.5 (33.3, 78.3)/58.0 (38.5, 90.8)	*0.153*
Stage (I-II, III-IV)	201/434	43.5 (30.1, 63.0)/55.6 (35.4, 88.1)	<***0.001***
Lymphatic metastasis (absent/present)	233/402	43.3 (30.7, 62.5)/57.1 (35.7, 92.8)	<***0.001***
Metastasis (absent/present)	470/165	45.3 (30.9, 70.6)/72.6 (43.3, 131.0)	<***0.001***

All values are presented in median and quartile intervals. The results of *p* < 0.05 were highlighted in bold italics.

### Correlations Between the Plasma Levels of HSP90α Protein With Routine Tumor Markers and Immune Cells

**FIGURE 2:**
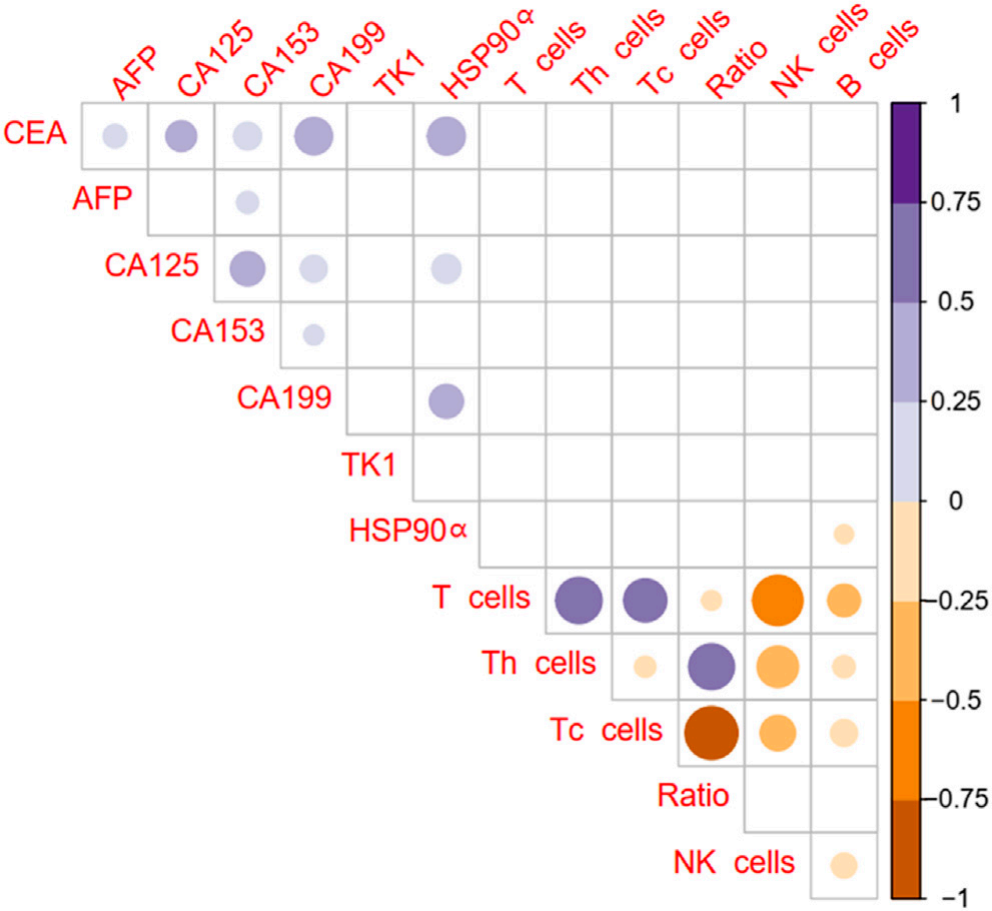
Correlation of plasma HSP90α protein levels with routine tumor markers and immune cells in colorectal cancer patients (Pearson analysis). Blue color represents a positive correlation, brown color represents a negative correlation, and the shade of color represents the degree of correlation. The results with *p < 0.05* were filled with color and display in the diagram.

The correlations between tumor markers and immune cells percentage were showed in Figure 2. There was no statistical correlation between common tumor markers CEA, AFP, CA125, CA153, CA199, TK1 and immune cells in patients with colorectal cancer (all *p > 0.05*, Figure 2). Plasma HSP90α protein levels were positively related to CEA, CA125, CA199 and negatively related to B lymphocyte percentage (all *p < 0.05*, Figure 2).

### Univariate and Multivariate Analysis for Factors Associated With Metastasis in Colorectal Cancer

**FIGURE 3:**
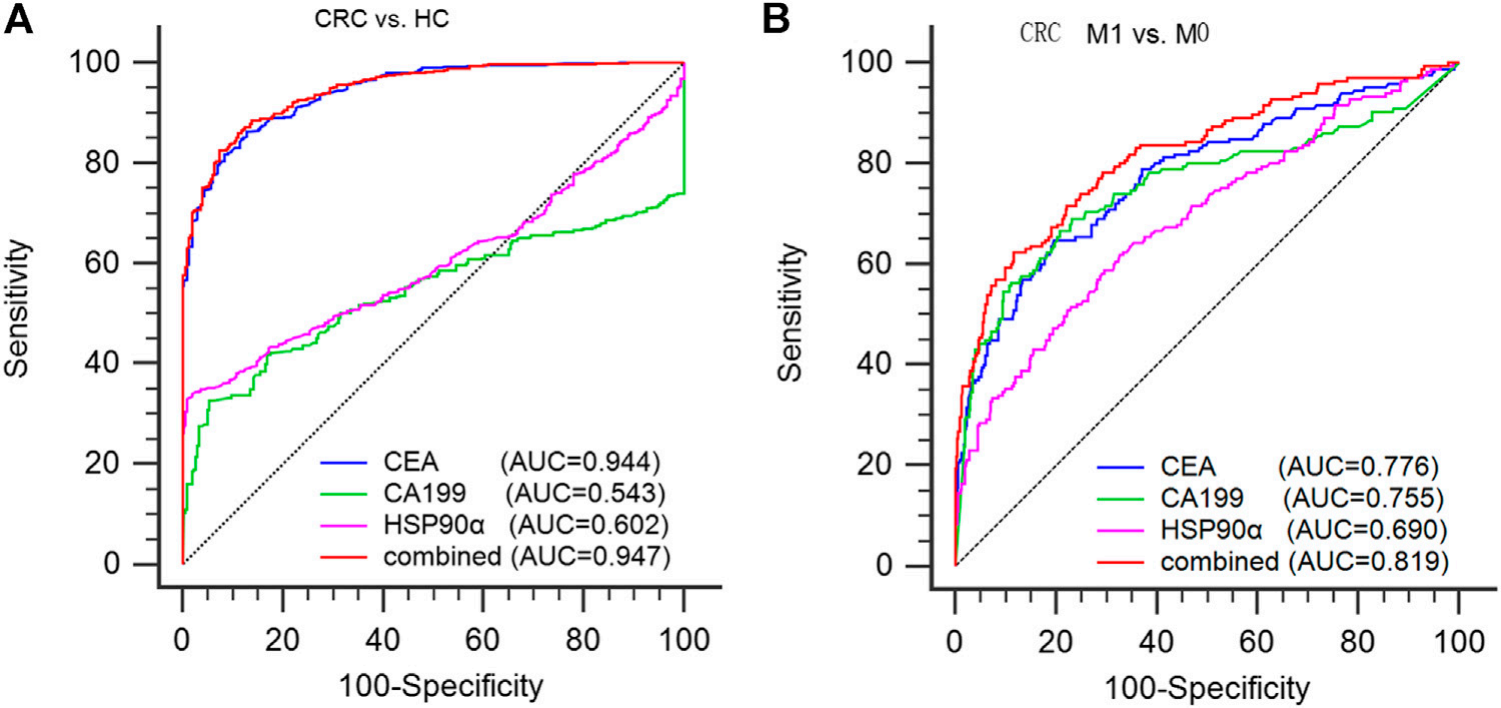
diagnostic ability of serum CEA, CA199 and plasma HSP90α in colorectal cancer. **(A)** Using healthy donors as controls to evaluate the efficacy of CEA, CA199, HSP90α and the panel in the diagnosis of colorectal cancer; **(B)** Using M0 patients as controls to evaluate the efficacy of CEA, CA199, HSP90α and the panel for distinguishing the presence of distant metastasis from CRC patients. M0: no distant metastasis; M1: distant metastasis.

The univariate and multivariate analysis for factors associated with metastasis in colorectal cancer were showed in Table 3. The univariate analysis showed that the biomarkers as CA125, CEA, CA199 and HSP90α were associated with metastasis in colorectal cancer patients, and the multivariate analysis revealed that the biomarkers as CEA, CA199 and HSP90α were independent risk factor for distant metastasis in patients with colorectal cancer (all *p* < 0.001). A nomogram for predicting the presence of metastasis in patients with colorectal cancer was showed in Figure 3.

**TABLE 3:** Univariate and multivariate analysis for factors associated with metastasis in colorectal cancer.

	Univariate analysis OR (95%CI)	*p* value	Multivariate analysis OR (95%CI)	*p* value
Gender	0.789 (0.540,1.154)	*0.223*	*—*	*—*
Age	0.902 (0.633, 1.286)	*0.569*	*—*	*—*
T cells	0.988 (0.970, 1.005)	*0.174*	*—*	*—*
Th cells	0.994 (0.973, 1.015)	*0.564*	*—*	*—*
Ts cells	0.988 (0.962, 1.016)	*0.399*	*—*	*—*
Ratio	1.011 (0.842, 1.213)	*0.909*	*—*	*—*
NK	1.002 (0.980, 1.024)	*0.872*	*—*	*—*
B cells	0.999 (0.965, 1.034)	*0.940*	*—*	*—*
CA153	1.024 (0.999, 1.050)	*0.063*	*—*	*—*
AFP	1.063 (0.991, 1.141)	*0.089*	*—*	*—*
TK1	0.997 (0.876, 1.135)	*0.964*	*—*	*—*
CA125	1.010 (1.005, 1.016)	*<0.001*	1.695 (1.000, 1.008)	*0.090*
CEA	1.008 (1.005, 1.011)	*<0.001*	3.647 (1.003, 1.008)	*<0.001*
CA199	1.003 (1.002, 1.004)	*<0.001*	5.454 (1.001, 1.003)	*<0.001*
HSP90α	1.013 (1.010, 1.017)	*<0.001*	3.935 (1.004, 1.012)	*<0.001*

The *p* values were shown in italics.

### The Diagnostic Efficacy of Plasma HSP90α in Colorectal Cancer and its Ability to Distinguish Colorectal Cancer Patients With Distant Metastasis

**FIGURE 4:**
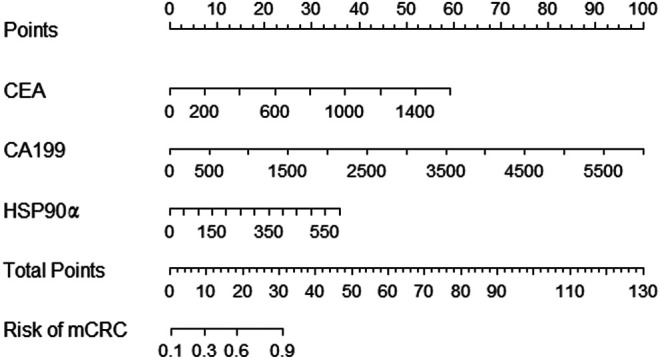
Nomogram for predicting the presence of metastasis in patients with colorectal cancer.

ROC analysis was conducted to assess the diagnostic ability of plasma HSP90α for colorectal cancer and results were showed in Figure 4. Using serum CEA, CA199 and plasma HSP90α levels of healthy donors as control group, the serum CEA has significant advantages in colorectal cancer diagnosis with a cut-off value 1.77 ng/ml (AUC = 0.944, sensitivity 86.3%, specificity 87.12%, Figure 3A). However, the plasma HSP90α levels showed a poor performance in colorectal cancer diagnosis with a cut-off value 69.1 ng/ml (AUC = 0.602, sensitivity 33.1%, specificity 99.0%, Figure 3A). Patients with colorectal cancer were divided into M0 and M1 groups according to the absence or presence of distant metastasis. Using the M0 group as controls, the AUCs of CEA, CA19-9, HSP90α and the panel for distinguishing the presence of distant metastasis from CRC patients were 0.776, 0.755, 0.690, 0.819, respectively (Figure 3B). A nomogram for predicting the presence of metastasis in patients with colorectal cancer was showed in Figure 4, higher total score based on the sum of the assigned numbers for each of the factors in the nomogram was associated with a high risk of mCRC.

### Correlations Between Concentrations of HSP90α, CEA and CA19-9 and PFS in CRC Patients

**FIGURE 5:**
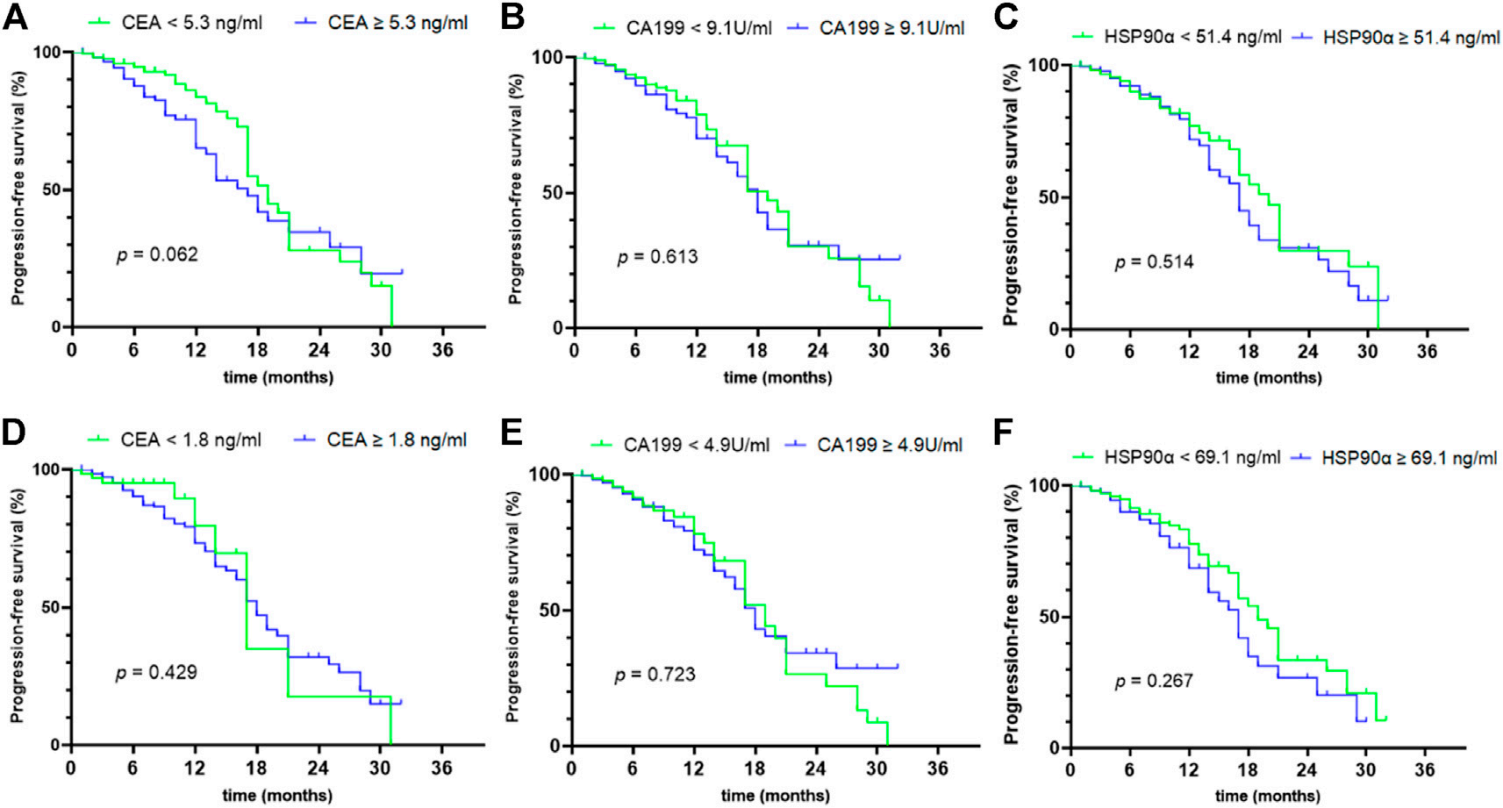
prognostic value of initial concentrations of CEA, CA199 and HSP90α in colorectal cancer. **(A–C)** The prognostic values of initial concentrations of CEA, CA199 and HSP90α were analyzed by grouping based on median value; **(D–F)** The prognostic values of initial concentrations of CEA, CA199 and HSP90α were analyzed by grouping based on the cut-off value of ROC analysis in Figure 3A.

Of 635 colorectal cancer patients, 454 cases had complete follow-up data and received standardized treatment. The median follow-up time was 7 months (range from 1 to 32 months). Survival analysis was conducted based on the median of CEA, CA19-9 and HSP90α levels in all colorectal cancer patients and the cutoff values calculated according to Yuden index respectively, and the results are shown in Figure 5. The initial concentrations of CEA, CA19-9 and HSP90α were not correlated with PFS in colorectal cancer (all *p* > 0.05, Figure 5).

### HSP90α Expression in TCGA Colorectal Cancer

**FIGURE 6:**
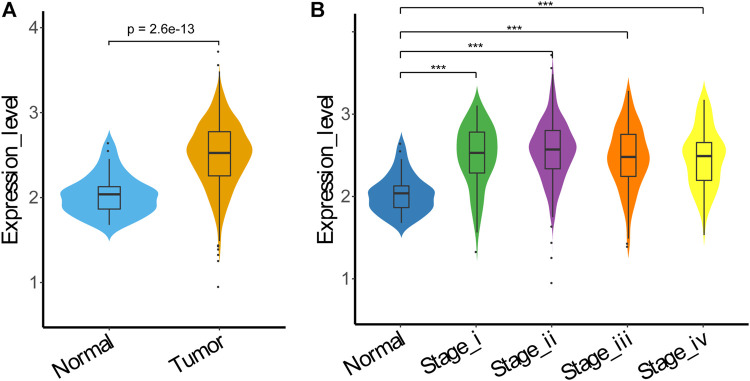
HSP90α expression in TCGA COAD. **(A)** HSP90α expression in TCGA COAD samples from normal tissue (*n* = 40) or tumor tissue (*n* = 458); **(B)** HSP90α expression in TCGA COAD samples from normal tissue (*n* = 40) or different tumor stage tissue (stageⅠ: *n* = 78, stageⅡ: *n* = 183, stage III: *n* = 132, stage IV: *n* = 65). Data were analyzed using Wilcoxon Rank Sum test.

To further explore the expression status of HSP90α in colon adenocarcinoma (COAD), we analyzed the expression data of HSP90α in COAD samples using data retrieved from The Cancer Genome Atlas (TCGA), and found that the expression levels of HSP90α in tumor samples were significantly higher than these from normal samples (*p* < 0.001), as shown in Figure 6A. The expression levels of HSP90α were further compared in samples from different stages of tumor, but no significant difference was found, although the expression levels of HSP90α in samples from all stages were higher than these from normal samples, as shown in Figure 6B.

## Discussion

HSP90α, which has evolved for almost 3.5 billion years, is a member of the HSP90 family, a conserved and essential molecular chaperone, can be translocated to the cell surface and secreted into the extracellular space by cancer cells (Frydman, 2001; Eustace et al., 2004). The secreted HSP90α was associated with tumor development and prognosis, especially with cancer metastasis (Passarino et al., 2003; Eustace et al., 2004; Chang et al., 2009). Previously studies have showed that plasma HSP90α is an excellent biomarker for the diagnosis of lung cancer and liver cancer (Jhaveri et al., 2014; Shi et al., 2014; Fu et al., 2017). Meanwhile, plasma HSP90α levels were significantly higher than healthy controls in other cancers, but the diagnostic efficiency was insufficient, such as gastric cancer, breast cancer, nasopharyngeal carcinoma and colorectal cancer (Kasanga et al., 2018; Liu et al., 2019; Lin et al., 2020; Zhang et al., 2020). In current study, the plasma HSP90α levels was significantly higher in CRC patients than in healthy controls and consistent with previously reported results (Kasanga et al., 2018). Additionally, this current study finds that plasma HSP90α levels were associated with gender and disease progression as stage, lymphatic metastasis and distant metastasis in colorectal cancer patients. Plasma HSP90α levels were also observed in breast, liver and nasopharyngeal cancers to be closely correlated with disease stage and distant metastasis, but not with gender (Fu et al., 2017; Wei et al., 2020; Mao et al., 2021). Therefore, we speculate that the gender differences in HSP90α levels may be influenced by hormones. Multivariate statistical analysis also showed that the serum CEA, CA199 and plasma HSP90α are independent risk factors for distant metastasis, respectively. The Nomogram prediction of distant metastases in combination with CEA, CA199, and HSP90α can provide evidence for clinical treatment plans that may lead to better patient outcomes.

In correlation analysis, the common tumor makers CEA, AFP, CA125, CA153, CA199 showed no statistical correlation with immune cells. But it is worth noting that the plasma HSP90α levels was highly correlated with common tumor biomarkers as CEA, CA125, CA199 and immune cells as B lymphocyte that indicates that the expression of plasma HSP90α is closely related to the regulation of immune function. However, the mechanism of the association between plasma HSP90α and immune cells in the occurrence and progression of cancer is still unknown. It is well known that cancer is also an immune-related disease. At present, the detection of immune cells is one of the commonly used methods to monitor the body immunity in clinical practice. Previous studies have reported that NK cells percentage and B lymphocyte are associated with survival in CRC patients (Berntsson et al., 2016; Tang et al., 2020). However, the plasma HSP90α levels were failed to find the significant prognosis value in colorectal cancer patients in this study. It's worth noting that the endpoint of most of the previous studies was overall survival (OS), while the endpoint of this study was PFS. Therefore, we speculated that although plasma HSP90α levels is not associated with PFS, it may be associated with OS in colorectal cancer patients and further research is needed to confirm this hypothesis.

Plasma HSP90α is overexpress and increases multi-malignant phenotypes including chemoresistance to cisplatin as well as metastatic potentials in various types of cancers (Eustace et al., 2004; Chang et al., 2009; Zuehlke et al., 2015). Therefore, targeted treatment with HSP90α inhibitors offer interesting perspectives for the treatment of cancers. Previous studies have showed that cellular secretion of HSP90α from colorectal cancer cells was enhanced after serum starvation, and secreted HSP90α could be used to stimulate migration and invasion of other non-serum-starved cells, and the mechanism might be that secreted HSP90α acts through TCF12 expression to enhance CRC cell spreading (Chen et al., 2010; Chen et al., 2013). Currently, approximately 20 of these inhibitors have undergone clinical evaluation (Jhaveri et al., 2012; Jhaveri et al., 2014). A recent study reported that HSP90α inhibition sensitized immune-refractory tumor to adoptive T cell transfer as well as PD-1 blockade, and re-invigorated the immune cycle of tumor-reactive T cells (Song et al., 2020). From the results of this study, we know that the expression of plasma HSP90α is related to immune cells. Therefore, the use of HSP90α inhibitors alone or in combination with chemoradiotherapy or immunotherapy may be one of the treatment methods to improve the prognosis of metastatic colorectal cancer. At the same time, dynamic observation of changes of in plasma HSP90α levels may be one of the monitoring methods for the therapeutic efficacy of HSP90α inhibitors.

## Conclusion

The present study demonstrated that plasma Hsp90α was remarkable higher in colorectal cancer and correlated with common tumor biomarkers and immune cells. Plasma Hsp90α levels were associated with gender, stage, lymphatic metastasis and distant metastasis but a poor diagnosis performance in colorectal cancer. Meanwhile, the current study failed to show a prognostic value for plasma HSP90α in colorectal cancer.

## Data Availability

The original contributions presented in the study are included in the article/supplementary material, further inquiries can be directed to the corresponding authors.
